# THE IMPACT OF SLEEP ON MINDFULNESS, WELL-BEING, AND DEPRESSIVE SYMPTOMATOLOGY IN MIDDLE TO OLDER AFRICAN AMERICANS

**DOI:** 10.1093/geroni/igac059.1889

**Published:** 2022-12-20

**Authors:** Folasade Ilori, Sanaz Dabiri, Mara Ramirez-Ruiz, George Daniel, Robert Head, Regina Wright, Denee Mwendwa

**Affiliations:** Howard University, Washington, District of Columbia, United States; Howard University, Washington, District of Columbia, United States; Howard University, Washington, District of Columbia, United States; Howard University, Washington, District of Columbia, United States; Howard University, Washington, District of Columbia, United States; University of Delaware, Newark, Delaware, United States; Howard University, Washington, District of Columbia, United States

## Abstract

The decline in physical and mental well-being is common with age. The negative impact of poor quality of sleep and depressive symptomatology contributes to the decline in well-being among middle to older adults. African Americans are more likely to report a decrease in well-being as they age. They are also disproportionately affected by poorer sleep quality and may be at greater risk for depressive symptomatology. Dispositional mindfulness is linked to improved well-being. Research has shown dispositional mindfulness is related to adaptive health behaviors, improved sleep quality, and reduced negative affect. Mindfulness allows engagement of early regulation of intense emotional responses because of nonjudgmental acceptance of thoughts and emotions. Therefore, this study aims to examine the impact of mindfulness on physical/mental well-being and depressive symptomatology through sleep. 131 African American participants were recruited for the study, with an average age of 58 years. The results showed a significant effect of mindfulness on well-being and depressive symptoms. Additionally, the impact of mindfulness on mental and physical well-being was mediated by sleep quality (B = .7920, CI [.1482, 1.6702]) and sleep disturbance (B = 2.874, CI [.1582, 6.335]). The results also showed an indirect effect of mindfulness on depressive symptoms through sleep quality (B = –6.139 CI [-12.871, -.5298]). This study demonstrates the positive impact of mindfulness on well-being and depressive symptomatology. Sleep quality also plays an important role in the relationship between dispositional mindfulness and physical/mental well-being and depressive symptomatology in middle to older African Americans.

